# Genome‐wide association analyses reveal polygenic genomic architecture underlying divergent shell morphology in Spanish *Littorina saxatilis* ecotypes

**DOI:** 10.1002/ece3.5378

**Published:** 2019-08-09

**Authors:** Tony Kess, Elizabeth G. Boulding

**Affiliations:** ^1^ Department of Integrative Biology University of Guelph Guelph ON Canada; ^2^ Present address: Fisheries and Oceans Canada St. John's NL Canada

**Keywords:** genome‐wide association, genomic architecture, geometric morphometrics, *Littorina saxatilis*

## Abstract

Gene flow between diverging populations experiencing dissimilar ecological conditions can theoretically constrain adaptive evolution. To minimize the effect of gene flow, alleles underlying traits essential for local adaptation are predicted to be located in linked genome regions with reduced recombination. Local reduction in gene flow caused by selection is expected to produce elevated divergence in these regions. The highly divergent crab‐adapted and wave‐adapted ecotypes of the marine snail *Littorina saxatilis* present a model system to test these predictions. We used genome‐wide association (GWA) analysis of geometric morphometric shell traits associated with microgeographic divergence between the two *L. saxatilis* ecotypes within three separate sampling sites. A total of 477 snails that had individual geometric morphometric data and individual genotypes at 4,066 single nucleotide polymorphisms (SNPs) were analyzed using GWA methods that corrected for population structure among the three sites. This approach allowed dissection of the genomic architecture of shell shape divergence between ecotypes across a wide geographic range, spanning two glacial lineages. GWA revealed 216 quantitative trait loci (QTL) with shell size or shape differences between ecotypes, with most loci explaining a small proportion of phenotypic variation. We found that QTL were evenly distributed across 17 linkage groups, and exhibited elevated interchromosomal linkage, suggesting a genome‐wide response to divergent selection on shell shape between the two ecotypes. Shell shape trait‐associated loci showed partial overlap with previously identified outlier loci under divergent selection between the two ecotypes, supporting the hypothesis of diversifying selection on these genomic regions. These results suggest that divergence in shell shape between the crab‐adapted and wave‐adapted ecotypes is produced predominantly by a polygenic genomic architecture with positive linkage disequilibrium among loci of small effect.

## 1 INTRODUCTION

Local adaptation to ecological conditions is the central mechanism driving reproductive isolation during ecological speciation (Schluter, 2001). Important to understanding this process is uncovering the genomic changes underlying evolution of adaptations contributing to reproductive isolation (Coyne & Orr, 2004; Hoban et al., 2016). The identification of speciation genes and their associated genome‐wide patterns of differentiation can aid in better understanding how and when local adaptation can give rise to the differentiation of species (Nosil & Schluter, 2011). Research on the “how” of species differentiation at the genetic level has been aided by development of models describing differentiation at a genomic scale, in which initially divergent genomic regions house those genes involved in early local adaptation (Wu, 2001). As divergence progresses, these genomic regions of reduced gene flow increase in size, resulting in a mosaic pattern of heterogeneous differentiation when comparing genomes of speciating lineages (Nosil, Funk, & Ortiz‐Barrientos, 2009; Via & West, 2008). The homogenizing effect of ongoing gene flow during this process may select for genomic architectures that minimize recombination and reduce gene flow, through increased establishment probabilities of mutations that produce larger phenotypic effect (Griswold, 2006; Yeaman & Whitlock, 2011). Loci responsible for locally adapted traits are thus predicted to localize primarily in a few linked regions of the genome that exhibit elevated patterns of differentiation relative to the genomic background (Via, 2012; Wu, 2001).

However, genome‐wide architectures underlying ecological speciation have been identified in some systems (Keller et al., 2013; Lawniczak et al., 2010), and highly divergent genomic regions may not always correspond to ecologically relevant loci (Cruickshank & Hahn, 2014; Ravinet et al., 2017). Polygenic models of local adaptation with gene flow have also demonstrated that adaptation and speciation may be caused by coordinated action of many loci of small effect (Le Corre & Kremer, 2012; Flaxman, Wacholder, Feder, & Nosil, 2014; Yeaman, 2015). Subtle shifts in allele frequency occurring between trait‐associated loci and the rest of the genome are predicted in these scenarios, and models have demonstrated the role of genome‐wide linkage in enabling divergence (Flaxman et al., 2014). Identifying the extent that genomic architectures predicted in models of speciation with gene flow correspond to observed patterns poses an ongoing challenge to understanding speciation genomics (Seehausen et al., 2014).

In this study, we use restriction‐associated DNA sequencing to determine whether the genomic architecture of adaptive traits key in ecological speciation follows patterns predicted in simple or polygenic models of local adaptation. Identification of the genomic architecture underlying ecological speciation can be conducted through tests of genomic divergence, paired with quantitative genetic methods, such as genome‐wide association (GWA), that identify associations between ecologically divergent traits and causal loci or loci in close physical linkage with them (Brennan et al., 2018; Stinchcombe & Hoekstra, 2008), revealing ecologically important quantitative trait loci (QTL). Combining tests of genomic divergence and GWA conducted within species undergoing ecological speciation can help delineate whether speciation with gene flow is initiated by highly divergent genomic regions, or can instead be produced by coordinated action of polygenic loci without pronounced differences in allele frequencies, and can direct further characterization of the molecular mechanisms contributing to locally adapted traits (Barrett & Hoekstra, 2011). Additionally, trait association methods can help disentangle the role of selective, demographic and structural factors in driving genomic differentiation.

GWA methods provide the opportunity to study naturally occurring genomic variation and its association with locally adapted traits. Although GWA shows promise in identifying loci involved in local adaptation putatively neutral genetic divergence occurring as genomes become structured within populations or ecosystems may produce false‐positive associations. Association models that fail to correct for this divergence risk falsely associating traits with neutral loci differentiated following decreases in genetic exchange, requiring methods to account for genetic structuring (Vilhjálmsson & Nordborg, 2013; Voight & Pritchard, 2005). Genomic divergence produced by local adaptation poses greater difficulty in identifying true associations between traits and markers, as correction for patterns of local differentiation may also reduce signals of true association corresponding to locally adapted loci (Atwell et al., 2010; Platt, Vilhjálmsson, & Nordborg, 2010; Johnston et al., 2014; Lotterhos & Whitlock, 2015). There remains debate over appropriate methods of accommodating population structure while detecting true trait‐associated loci in populations with complex divergence histories (Forester, Lasky, Wagner, & Urban, 2018; Hoban et al., 2016; Vilhjálmsson & Nordborg, 2013). In this study, we compare results from multiple proposed methods used in correcting for population structure and relatedness, using both single‐locus and polygenic detection methods to identify the genomic basis of local adaptation in the marine snail *Littorina saxatilis*.

*Littorina saxatilis* is considered a model system for the study of ecological speciation because it forms pairs of locally adapted “crab” and “wave” ecotypes across shared intertidal ranges in rocky beach sites across the Northeast Atlantic (Johannesson et al., 2010; Rolán‐Alvarez, Austin, & Boulding, 2015). These ecotypes show repeatable, parallel adaptation to crab predation or wave action in habitats within the intertidal zone (Butlin et al., 2014) (Boulding, Rivas, González‐Lavín, Rolán‐Alvarez, & Galindo, 2017; Johannesson, Johannesson, & Rolán‐Alvarez, 1993). Adaptive shell traits are thought to have evolved in situ despite ongoing gene flow (Butlin et al., 2014; Galindo, Martínez‐Fernández, Rodríguez‐Ramilo, & Rolán‐Alvarez, 2013; Kess, Galindo, & Boulding, 2018; Rolán‐Alvarez et al., 2004) and have been shown to mediate partial reproductive isolation between the two ecotypes with respect to shell size (Rolán‐Alvarez et al., 2015), implicating shell size divergence as a “magic trait” facilitating both local adaptation and assortative mating (Boulding et al., 2017; Galindo, Cacheda, Caballero, & Rolán‐Alvarez, 2019; Johannesson et al., 2010; Servedio, Doorn, Kopp, Frame, & Nosi, 2011).

Although the importance of antipredator shell traits in facilitating divergence among ecotypes has been demonstrated in *L. saxatilis* (Boulding et al., 2017), little is known about the genomic architecture underlying shell morphology (Rolán‐Alvarez et al., 2015). Studying the genomic basis of shell traits can provide insight into the genomic architecture that has produced local adaptation observed among *L. saxatilis* ecotypes despite ongoing gene flow (Johannesson, Butlin, Panova, & Westram, 2018). Comparisons using a smaller set of dominant markers have found partial overlap with outlier loci, but detection of individual trait‐associated loci using high‐resolution genomic datasets within this system has remained elusive (Westram, Panova, Galindo, & Butlin, 2016), despite repeated identification of extensive genome‐wide divergence between ecotypes (Kess et al., 2018; Ravinet et al., 2016; Westram et al., 2016), underscoring the importance of investigating the phenotypic contributions of genomic regions of divergence with a variety of GWA methods. Here, we use a set of genome‐wide SNPs from three sampling sites, spanning two glacial lineages and paired with individual shell phenotypes. This dataset allowed us to study the genomic architecture underlying shell divergence at a broad geographic scale, across the Galician *L. saxatilis* range.

In this study, we used GWA to uncover the genomic architecture underlying variation in shell morphology between *L. saxatilis* ecotypes. We address three main goals in this study: (a) geometric morphometric characterization of shell morphology variation among crab and wave ecotypes and phenotypically intermediate individuals, to identify shell shape differences reflecting divergence between ecotypes for testing in genome‐wide association; (b) genome‐wide association analysis of each shell trait using 4,066 SNP markers developed using double‐digest restriction‐associated DNA sequencing (ddRAD); and (c) testing predictions for the evolution of genomic architecture during adaptation with gene flow, through identifying whether QTL exhibit elevated linkage or correspond to highly divergent genomic regions.

## 2 MATERIALS AND METHODS

### 2.1 Sample collection and genotyping

Genotyped samples used in this study were obtained from a previous ddRAD study of *L. saxatilis* crab ecotypes from the upper intertidal zone, wave ecotypes from the lower intertidal zone, and phenotypically intermediate samples with intermediate banding and riding shell trait combinations from the mid‐shore of the intertidal zone. Samples were collected from three sites on the North and Northwest coasts of Spain (Kess et al., 2018): Silleiro (long −8.90°, lat 42.13°), Corrubedo (long −9.10°, lat 42.60°), and Burela (long −7.36°, lat 43.67°). Library preparation for ddRAD was carried out using the protocol described in Kess, Gross, Harper, and Boulding (2016). Using Stacks 1.40 (Catchen, Amores, Hohenlohe, Cresko, & Postlethwait, 2011), we generated a panel of 4,066 SNP loci genotyped in a minimum of 70% of 477 sequenced individuals that also passed filtering using VCFtools 0.1.12b (Danecek et al., 2011) to exclude loci that deviated from Hardy–Weinberg equilibrium in all population samples at a Bonferroni‐corrected *α* of 0.05/number of tested SNPs.

### 2.2 Shell imaging and weight

We removed all tissue from shells during DNA extraction using forceps and 70% ethanol. A total of 574 empty shells from all collected individuals that were processed for DNA extraction, including the 477 successfully sequenced individuals, were photographed to record shell phenotypes, using a digital camera under magnification using a dissection microscope. Prior to imaging, shells were individually weighed and each weight was recorded. Shells were then positioned on a base of dental wax such that shell aperture was parallel to the camera lens, with the axis of coiling and spire on the same vertical axis in the photograph. Each shell was photographed against a transparent plastic size standard grid with 1mm x 1mm squares to enable size and magnification correction in geometric morphometric analysis.

### 2.3 Geometric morphometric analysis

**Figure 1:**
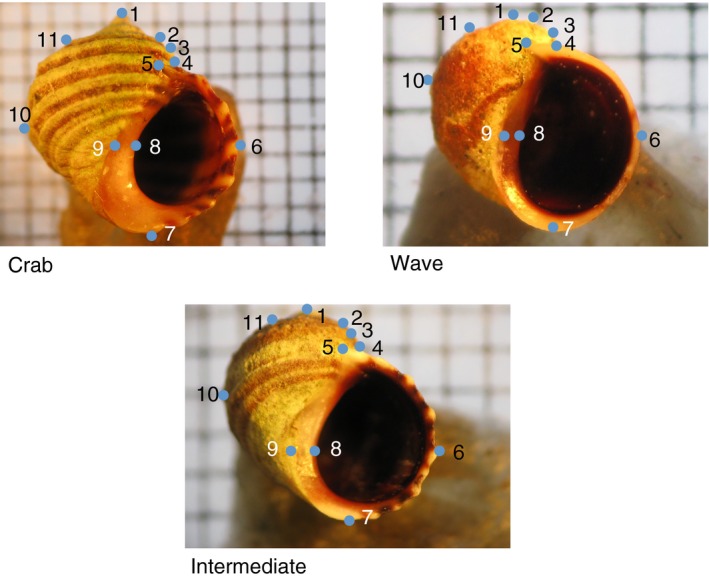
Positions of 11 geometric morphometric landmarks developed by Carvajal‐Rodríguez et al. (2005) on shell features of crab and wave *Littorina saxatilis* ecotypes and intermediate individuals from the mid‐shore

To characterize shell morphology of all sampled individuals, we used geometric morphometric landmarks developed by Carvajal‐Rodríguez, Conde‐Padin, and Rolán‐Alvarez (2005) to score *x*‐ and *y*‐coordinates of 11 of the original 12 landmark locations on each shell (Figure 1) and 1 mm size standards in each photograph, using tpsDig 2.17 (Rohlf, 2013). The tenth landmark described in Carvajal‐Rodríguez et al. (2005) was removed due to low repeatability. Shell landmark consistency was checked by repeating scoring of one representative crab ecotype, wave ecotype, and phenotypically intermediate photograph five times by two separate technicians, followed by comparison of landmark point separation in tpsRelw 1.53 (Rohlf, 2015), and adjustment of landmark scoring criteria used across photographs prior to landmark scoring of the total sample. Variation due to shell rotation in photographs was checked by repeating photographs of the same representative ecotypes and visualizing point separation, followed by standardization of photography protocol until landmark separation within scored individuals was minimized.

Aligned landmarks, centroid size, and nonuniform components of shape variation were calculated in tpsRelw. We adjusted raw landmark *x* and *y* values for each sample using generalized Procrustes analysis to retain only shape information from landmark positions by removing variation due to size, position, and rotation (Rohlf & Slice, 1990). This operation was carried out in tpsRelw by removing variation unrelated to shape through superimposing individual landmark values onto a consensus shape, calculated by minimizing the differences in sums of squared landmark distances between the same landmarks in each sample. The consensus shape was calculated from the complete set of all photographed shells. Centroid size, used as an estimate of shell size, was calculated in tpsRelw during alignment and consensus shape estimation as the square root of the sum of the squared distances between the average *x*‐coordinate and average *y*‐coordinate of all landmarks for each individual snail (the centroid) and the corresponding coordinates of each of its landmarks (Bookstein, 1991). tpsRelw was also used to calculate eight partial warps corresponding to nonuniform shape changes between specimens at different spatial scales, and partial warp scores which describe the orientation and direction of shape change at each partial warp away from the reference for each individual (Zelditch, Swiderski, Sheets, & Fink, 2004). Relative warps, which characterize separate, uncorrelated major axes of shape variation within the sample, were obtained by carrying out principal component analysis on partial warp scores for each individual (Rohlf, 1993). Changes of shell shape corresponding to changes in relative warp scores for the first two principal components were visualized on deformation grids using tpsRelw.

### 2.4 Estimation of shell morphology divergence between ecotypes

We then characterized the extent that phenotypic variation in shell traits corresponded to differences between ecotypes. We used RSTUDIO 0.99.893 (RStudio Team, 2015) using R version 3.3.3 (R Core Team, 2017) to calculate ANCOVA models to quantify the contribution of ecotype divergence to shape variation between individuals. To distinguish between the role of local selection within ecotypes or shared evolutionary history within populations in explaining different components of shell shape, we ran separate ANCOVAs, each using one of the scores for relative warps 1 to 5 (RW1‐5) as dependent variables. Interaction between the centroid size covariate was included to account for shell shape allometry previously identified in the Silleiro population studied by Carvajal‐Rodríguez et al. (2005). Variation attributable to shared selection on ecotypes, or shared differences in evolutionary history were identified by significant *p*‐values for each of these factors, below a Bonferroni‐corrected α value of 0.0083.

### 2.5 Genome‐wide association of shell morphology

#### 2.5.1 Single‐locus models

We used score tests implemented in the R package *GenABEL* 1.8–0 (Aulchenko, Ripke, Isaacs, & Duijn, 2007a) to conduct GWA analysis to identify genotype–trait associations for shell morphology traits across ecotypes and populations, using the panel of 4,066 SNP loci that had passed quality control. Separate GWA analyses were carried out for 11 *x* and *y* aligned landmark coordinates, relative warps 1 and 2, shell weight, and centroid size. Aligned landmarks were used rather than relative warps as the former may better correspond to localized expressed transcripts from homeotic and other genes during development (Boulding et al., 2008). To control for allometry, centroid size was included as a covariate in the linear models for all shape traits. A Bonferroni‐corrected α of 0.05/4066 tested SNPs was used to determine whether an association with a SNP was significant in each test.

To quantify population structure for correction in GWA models, we used principal coordinate analysis of genetic distances between individuals using 2405 putatively neutral SNPs. To produce this dataset, we used a conservative approach to remove SNPs exhibiting signatures of potential selection, removing all SNPs identified as potential outliers in at least one outlier test in Kess et al. (2018), or exceeding the 95th percentile of the genome‐wide *F*_ST _distribution, calculated in plink 1.9 (Chang et al., 2015). We then identified trait associations with SNPs using two models frequently used for controlling for population structure in GWA: principal coordinate and genomic kinship matrix corrections, using combinations of *GenABEL* functions described below (Hecht, Campbell, Holecek, & Narum, 2013; Lawson & Petren, 2017; McKown et al., 2014). These models identify correlations between each SNP and each phenotypic variable, after variation due to population structure has been controlled for by inclusion of either a genomic kinship matrix or principal components or coordinates that explain a significant proportion of population structure.

We used the *ibs* function in *GenABEL* to calculate a genomic kinship matrix (K) containing pairwise identity‐by‐state (IBS) values estimated using all of the 2405 putatively neutral SNPs that were successfully genotyped for that pair of individuals (Aulchenko et al., 2007a). We then produced a distance matrix describing pairwise measures of genetic distance between individuals by subtracting 0.5 – K from each element. We then used classic multidimensional scaling on this matrix to calculate principal coordinates of genetic distance values between individuals, using the *cmdscale* function in R. Scree plots (Cattell, 1966) were used to visualize variation explained by each principal coordinate. We then included the top three principal coordinates which each explained > 5% of variation in genomic differentiation between individuals (*β_PC_*_1,_ *_PC_*_2,_ *_PC_*_3_) as covariates in a linear model predicting phenotype ($Y_{i})$ with intercept μ and random error term, *e* (PC model), similar to the model used in Price et al. (2006). Associations were detected between each shell trait and each SNP using a score test carried out by the *qtscore* function in *GenABEL*, which approximates the model below:

(1) $$\begin{array}{c} Y_{i}=\mu +\beta_{\text{SNP}}*\text{SNP}+\beta_{\text{PC1}}*PC1+\beta_{PC2}*PC2\\ +\beta_{PC3}*PC3+\beta_{\text{CS}}*\text{CS}+e_{i} \end{array}$$

We also conducted GWA using a two‐step linear mixed model using the genomic kinship matrix (K) to estimate the proportion of phenotypic similarity between individuals that can be attributed to their extent of shared common ancestry. This model follows the family‐based association model described in Chen and Abecasis (2007) and Aulchenko, Ripke, Isaacs, and Duijn (2007b), with genomic identity by state between individuals calculated directly from allele frequency, as in Kang et al. (2010). Individuals are presumed to have a phenotype value that is determined by population mean phenotype *µ*, plus the contribution of additive genetic effects $G_{i}$ and *j* covariates *X* with slope $\beta_{X}$. For the shape variables, we included centroid size as a covariate. The random additive polygenic effect $G_{i}$ is distributed with variance K $\sigma_{G}^{2}$, where K is the genomic kinship matrix calculated from 4066 SNP loci and $\sigma_{G}^{2}$ is the additive genetic variance. In the first step, the maximum likelihood estimated trait variation in an individual explained by additive genetic contributions of shared alleles, ${\hat{e}}_{i}$, and the covariate(s) is estimated using the *polygenic* function, calculated using the linear mixed model:

(2) $$Y_{i}=\mu +\sum_{j}\beta_{X}X+G_{i}+{\hat{e}}_{i}$$

A score test for each genotyped SNP was then performed using *mmscore* to approximate the fit of a second model. This model fits calculated residual values representing trait variation not explained by genome‐wide relatedness between individuals, or included covariates, as the dependent variable, using the following equation:

(3) $${\hat{e}}_{i}=\mu +\beta_{\text{SNP}}*\text{SNP}+e_{i}$$

We tested the second *mmscore* model using structured association (Pritchard, Stephens, Rosenberg, & Donnelly, 2000) (K:SA) with the dependent variable being residuals calculated from the *polygenic* model in Equation (4) including only centroid size as a covariate, as shown in the equation:

(4) $${\hat{e}}_{i}=\mu +\beta_{\text{SNP}}\left(\text{population}\right)*\text{SNP}+e_{i}$$

The coefficients of β were estimated separately for all populations (Silleiro, Corrubedo, Burela) and were then combined using a weighted average with the weight being the squared inverse of the standard error of β.

Score test values distribute approximately as a $\chi^{2}$ distribution (Chen & Abecasis, 2007), allowing SNP effect size ($r^{2}$) calculation from one‐degree‐of‐freedom $\chi^{2}$ test statistics obtained from the score test using the following formula:

(5) $$r^{2}=\frac{\chi^{2}}{n-2+\chi^{2}}$$

where *n* is the number of individuals genotyped per tested SNP.

### 2.6 Redundancy analysis

Multivariate methods have recently been utilized in identifying QTL in genotype–environment associations (Forester et al., 2018; Harrisson et al., 2017). These methods have the advantage of simultaneously accounting for multiple drivers of phenotypic or environmental divergence, and identifying polygenic trait architectures. Here, we used redundancy analysis in the R package *vegan* 2.4‐5 (Okansen et al., 2017) to identify trait‐associated loci by using morphometric variables as predictors, and identify SNPs with the greatest constrained variance explained by predictors as QTL. In the RDA model, we used all traits used in single‐locus GWA methods as predictors, excluding those that exhibited correlations greater than 0.7, and a matrix of complete, imputed genotypes for all 4066 SNP loci in each site as response variables. We imputed missing loci using LinkImpute (Money et al., 2015). Following the methodology of Forester et al. (2018), we identified QTL as those exhibiting RDA loadings greater than three standard deviations from the mean. SNP loadings were characterized for the first three canonical axes, which were selected based on visual inspection of Scree plots. Outlier SNPs were then correlated with each shell trait variable, and the strongest correlation for each SNP was used to group SNPs by traits driving their differentiation.

### 2.7 Characterization of linkage and differentiation

During adaptation with gene flow, loci contributing to phenotypic differentiation are predicted to exhibit elevated linkage disequilibrium (LD) and divergence (Flaxman et al., 2014; Schilling et al., 2018). We used plink 1.90b3.46 (Chang et al., 2015) to assess pairwise LD between all 4066 SNPs. We then compared pairwise *r* ^2^ among identified QTL with putatively neutral SNPs that were not significantly associated with shell traits, did not exhibit elevated differentiation based on *F*_ST_ values greater than the 95% quantile as calculated in plink, or were not previously identified as *F*_ST_ outliers using formal outlier tests in Kess et al. (2018). We compared *r* ^2^ for each class of SNP using a Mann–Whitney U test within high and low shore locations in each site and between ecotypes in each site, as in Schilling et al. (2018), and used a Mann–Whitney U test on *r* ^2^ values for QTL and putatively SNPs to assess the significance of LD differences.

To directly test the genomic distribution of QTL, we aligned our RAD loci containing QTL to the recently released draft genome from the Swedish crab ecotype of *L. saxatilis* (Westram et al., 2018). We extracted RAD loci with QTL from the Stacks consensus sequences using the *filterbyname* scripts in BBMap (Bushnell, 2016). Alignments were carried out with Burrows–Wheeler Aligner, using the bwa mem algorithm (Li, 2013). We then binned aligned contigs to their corresponding linkage group information (Westram et al., 2018). To test whether QTL exhibit nonrandom distribution among linkage groups, the proportion of QTL aligning to each linkage group was compared using a $\chi^{2}$ test.

To identify whether trait‐associated loci demonstrate strong signals of differentiation potentially due to divergent selection, we compared identified QTL with genomic regions exhibiting elevated differentiation based on *F*_ST_ values greater than the 95% quantile as calculated in plink, or previously identified as *F*_ST_ outliers using formal outlier tests in Kess et al. (2018). This significance threshold was selected to ensure all divergent loci or those significantly associated with population structure were included for comparison with trait‐associated SNPs. Following these analyses, we quantified the number of trait‐associated loci also identified as divergent loci.

## 3 RESULTS

### 3.1 Geometric morphometric analysis and sources of shape variation

**Figure 2:**
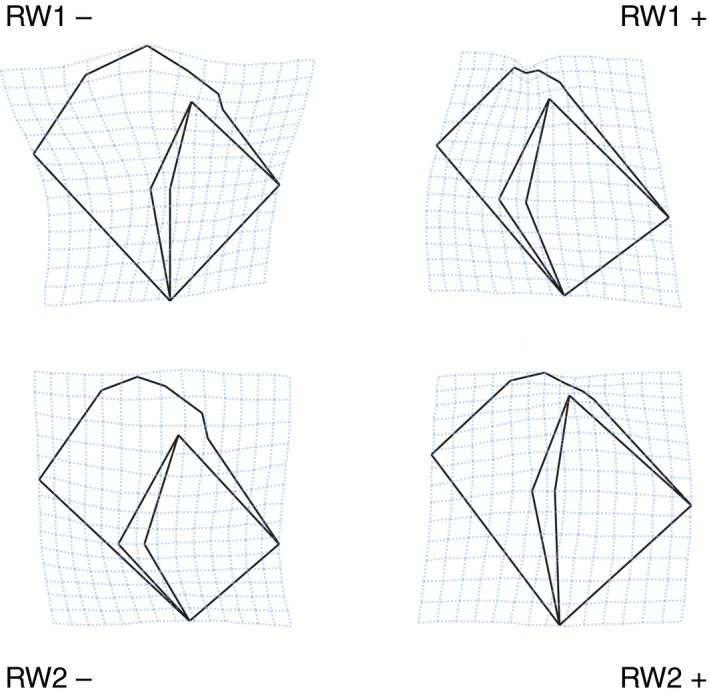
Deformation grids for relative warp 1 (RW1) and relative warp 2 (RW2) values calculated from 11 geometric morphometric landmarks, depicting shell shape change with change of relative warp value, and ecotype labels identifying shape change specific to each ecotype

We found that the majority (62.79%) of shape variation was explained by the first two relative warps which reflected differences in shell aperture and spire height. The first relative warp accounted for 43.16% (RW1) and the second accounted for 19.63% (RW2) of shell shape variation. We found five relative warps that individually explained greater than 5% of shell shape variation and cumulatively explained 84.30% of variation between individuals, of which RW3, RW4, and RW5 each respectively explained 8.79, 7.8, and 5.31% of total shape variation. Visualizing changes in shell shape with changing relative warp values using deformation grids revealed increases in RW1 values corresponding with increase in shell aperture size and reduction in spire height (Figure 2). We found a similar pattern of shape change for RW2, with increases in RW2 value commensurate with reduction in upper whorl size, and upward expansion of the shell aperture.

We found that interactive effects between ecotype and population factors were observed in all traits excluding RW3, indicating nonshared, population‐specific differences in ecotype shell shape (Table 1). Interaction between these factors and centroid size also made a significant contribution to explaining variation in the first two relative warps, indicating population‐specific allometric relationships for these shape variables, and suggesting ecotype‐specific developmental trajectories of shape change vary between populations.

**Table 1:** Significant factors (ecotype [E] and population [P]), covariate (centroid size [C]), and interaction effects in order of significance, and adjusted *R* ^2^ values associated with measures of shell shape in ANCOVA of relative warp and centroid size obtained from geometric morphometric analysis

Trait	Significant factors, covariates, and interactions	*R* ^2^
RW1	E × P × C (*p* = 0.0008)	0.699
RW2	E × P × C (*p* = 8.8 × 10^−6^)	0.3147
RW3	E (*p* = 6.2 × 10^−10^), C (*p* = 1.35 × 10^−5^), P (*p* = 0.008)	0.1388
RW4	E × P (*p* = 0.0001)	0.1001
RW5	E × P × C (*p* = 0.007)	0.1139
Centroid	E × P (*p* = 0.0003)	0.6982

### 3.2 Genome‐wide association analysis and genomic architecture of shell morphology

**Figure 3:**
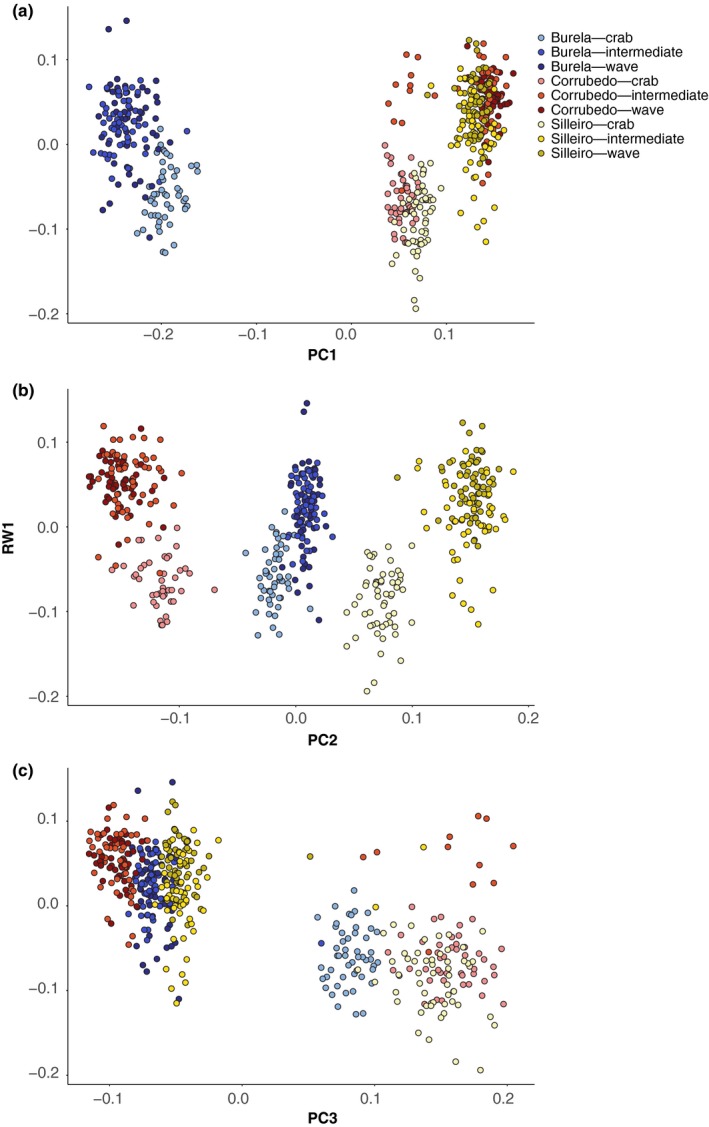
Plot of the first three principal coordinates of genetic distance between individuals from 2405 putatively neutral SNP loci, (a) PC1, (b) PC2, and (c) PC3, and relative warp 1 (RW1) describing shell shape differences calculated from 11 geometric morphometric landmarks

We observed polygenic bases of shell trait variation through identification of 216 QTL in genome‐wide association tests performed using single‐locus models in *GenABEL* and polygenic association using RDA, though associated locus identity varied depending on the method used to correct for population structure. We identified 97 QTL in the K:SA model, 57 in the PC model, and 82 using RDA (58 from RDA1, 8 from RDA2, 11 from RDA3). A small proportion of associations were common across pairwise comparisons between models: 15 QTL (0.108) were shared between the PCA and K:SA models, and four QTL (0.023) were shared between the RDA and K:SA models. However, only one QTL was shared between the RDA and PC models, and no QTL were shared among all three comparisons. The different methods of population structure correction used in each method likely account for these differences, as we found that individuals grouped by RW1 scores also clustered by PC3, whereas population differences were explained by PC1 and PC2 (Figure 3).

Consistent with a polygenic basis of shell variation, we found that the majority of identified loci in single‐locus models exhibited small effect sizes (~5%) for each trait (Table 2). However, a subset of large‐effect alleles were observed associated with X7, X9, and Y2 landmarks, RW1, all exceeding 10% variation explained; a single locus identified in the K:SA model explained 24% of centroid size variation.

**Table 2:** Number of SNP loci significantly associated with shell morphology traits and mean phenotypic variation explained (*r* ^2^) per SNP in a linear model with the first three principal coordinates of genotypic variation (PC model), and structured association conducted with a mixed linear model adjusted for population structure using the genomic kinship matrix (K:SA model), or exhibiting highest correlation with a shell morphology trait in redundancy analysis (RDA)

Trait	Associated SNPs		Max percentage phenotypic variation explained (*r* ^2^) by associated SNP		Median percentage phenotypic variation explained(*r* ^2^)/ associated SNP		SNPs exhibiting highest correlation with trait identified in RDA
	K:SA model	PC model	K:SA model	PC model	K:SA model	PC model	
X1	0	0	0	0	0	0	1
X2	4	0	5.9	0	5.1	0	0
X5	0	0	0	0	0	0	1
X7	23	21	10.2	8.0	5.9	4.9	2
X8	16	2	8.4	4.8	4.6	4.4	0
X9	30	26	15.1	8.8	6.0	5.5	10
X10	20	0	9.3	0	6.0	0	0
X11	6	0	6.6	0	5.5	0	0
Y1	0	4	0	5.9	0	5.1	0
Y2	17	10	10.7	5.3	5.8	4.9	1
Y3	0	0	0	0	0	0	1
Y4	1	9	4.9	9.0	4.9	4.8	0
Y5	0	3	0	8.2	0	5.4	0
Y6	9	0	8.0	0	5.5	0	1
Y7	0	7	0	5.1	0	4.7	0
Y10	0	0	0	0	0	0	1
Y11	8	0	8.2	0	6.6	0	0
RW1	20	0	11.1	0	6.4	0	0
RW2	3	15	6.4	7.9	5.0	5.2	0
Centroid	78	31	24.7	8.0	6.2	5.2	59

**Figure 4:**
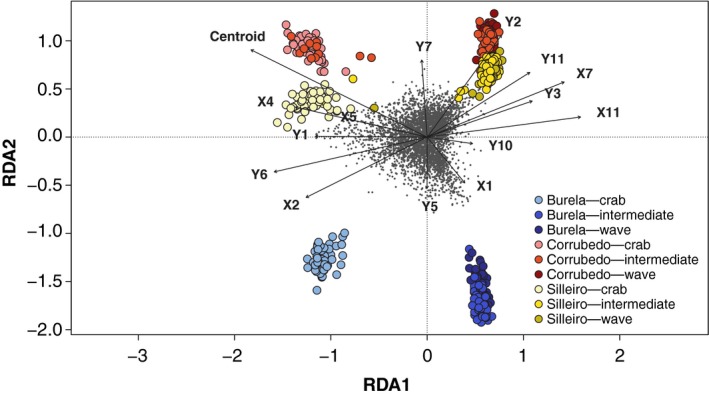
Plot of individual scores on the first canonical axes (RDA1 and RDA2) obtained from a constrained ordination of 4,066 SNP loci with 15 shell trait variables

We found that the majority of SNPs identified using RDA exhibited strongest association with centroid size (*n* = 59 SNPs), and landmarks Y3 (*n* = 10 SNPs) and X7 (*n* = 5 SNPs), supporting a role for aperture shape differences and shell shape facilitating divergence between ecotypes (RDA Figure). In contrast to the PC model, we observed separation of ecotypes followed by glacial lineages on the first two RDA axes when constrained by shell morphology, indicating shell morphology variation plays a role in genomic differentiation between ecotypes across sites (Figure 4).

### 3.3 Linkage disequilibrium

**Figure 5:**
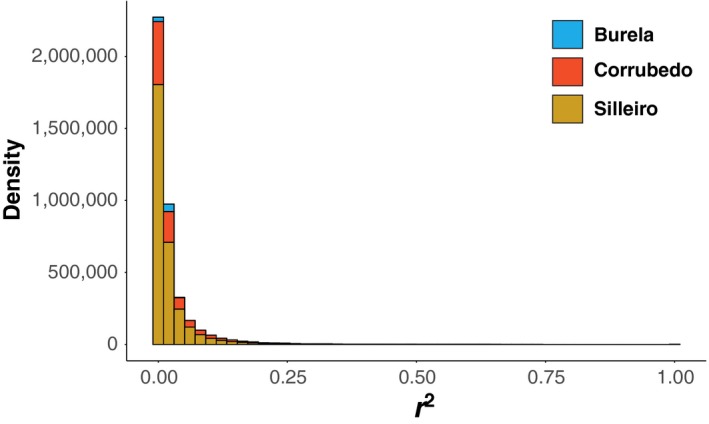
Distribution of linkage disequilibrium measured as pairwise *r* ^2^ between 4066 SNP loci, calculated separately in samples from Burela, Corrubedo, and Silleiro

We observed elevation of statistical LD among QTL, but identified a genome‐wide distribution of QTL overall, indicating genome‐wide selection on shell shape. We found that pairwise LD among all SNPs was low even between ecotypes (mean *r* ^2^ BR = 0.023, CO = 0.026, SI = 0.022; Figure 5) in each site, indicating low physical linkage of SNPs used in this study. To test for evidence of long‐distance linkage between trait‐associated loci, we compared levels of LD (*r* ^2^) within sampling locations and within ecotypes within locations (Table 3). We found that QTL exhibited significantly elevated LD within locations compared to the genome‐wide average for putatively neutral SNPs. Similarly, we found that within ecotypes within all sites, at least one ecotype exhibited signatures of long‐distance LD of QTL, indicated by slightly elevated *r* ^2^ values and shifted *r* ^2^ distributions of QTL relative to putatively neutral loci (Table 3).

**Table 3:** Linkage relationships of neutral loci and QTL in within‐site and within‐site‐and‐ecotype calculation of pairwise *r* ^2^ per SNP, and pairwise significance of compared SNPs evaluated using a Mann–Whitney U test

Site	Comparison	Putatively neutral *r* ^2^	QTL *r* ^2^	Mann–Whitney U test
SI	HL	0.016	0.102	W = 8,254,500,000, *p* < 2.2 × 10^−16^
	H	0.0253	0.0286	W = 12,113,000,000, *p* < 2.2 × 10^−16^
	L	0.0215	0.0355	W = 5,243,000,000, *p* = 0.704
CO	HL	0.0193	0.102	W = 13,481,000,000, *p* < 2.2 × 10^−16^
	H	0.0311	0.0314	W = 11,612,000,000, *p* = 0.0213
	L	0.0288	0.0284	W = 551,200,000, *p* = 7.852× 10^−8^
BR	HL	0.0185	0.0707	W = 13,542,000,000, *p* < 2.2 × 10^−16^ *p* = 8.47 × 10^−9^
	H	0.031	0.0322	W = 8,843,600,000, *p* = 0.00384
	L	0.0313	0.0302	W = 5,272,200,000, *p*‐value = 0.056

Alignment of RAD loci containing QTL to the *L. saxatilis* genome revealed that these loci were distributed across 196 contigs; 61 RAD loci aligned to contigs with linkage information. These 48 loci were found across 17 linkage groups, suggesting low physical linkage of QTL. Consistent with low physical LD between QTL, we did not find higher density of QTL across any aligned linkage group (χ^2^ = 14.66, *df* = 16, *p* = 0.5497).

### 3.4 Differentiation of QTL between ecotypes

**Figure 6:**
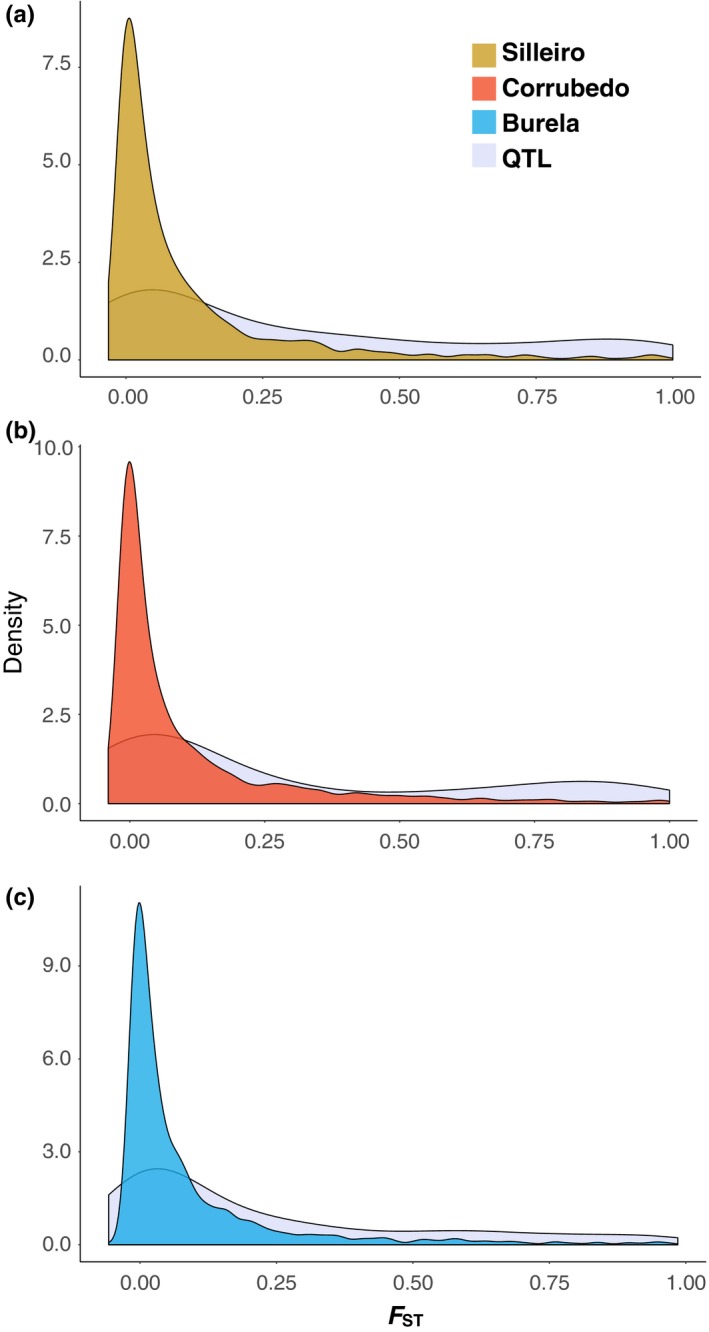
Comparison of *F*_ST_ distributions between 216 QTL and all 4066 SNPs in (a) Burela, (b) Corrubedo, and (c) Silleiro

Consistent with predictions from models of divergence with gene flow, we found partial overlap of divergent SNPs and QTL identified in this study. *F*_ST_ values of QTL were elevated relative to the genome‐wide distribution for all loci (Figure 6). Of the total set of identified QTL, 64 (0.296) exhibited overlap with loci in divergent genomic regions. This pattern of elevated divergence of QTL was driven by loci identified using RDA; 53 (0.646) QTL identified in RDA exhibited overlap with divergent regions, whereas only 11 (0.1134) QTL identified in the K:SA model overlapped with divergent regions, and only 3 QTL (0.052) identified in the PC model exhibited elevated divergence.

## 4 DISCUSSION

### 4.1 Polygenic size and shape divergence

Uncertainty remains over the extent that observed genomic architectures match those predicted to evolve during speciation with gene flow (Seehausen et al., 2014). Here, we used genome‐wide association of SNP loci with shell shape variation observed between *L. saxatilis* ecotypes to identify a polygenic, genome‐wide basis of ecotype divergence. Our dataset, sampled from three sites spanning two glacial lineages, enabled investigation of the genomic architecture underlying shell divergence between ecotypes across the geographic range of Galician *L. saxatilis* crab and wave ecotypes. We find evidence of: (a) polygenic architecture of differentiated shell shape and size traits, (b) elevated linkage of QTL associated with shell variation despite a genome‐wide distribution, and (c) partial overlap of divergent *F*_ST_ outlier regions associated with shape variation detected during genome‐wide association, suggesting increased rates of divergence at QTL underlying shell variation. Together these results from an empirical study of an emerging model system (Ravinet et al., 2017; Galindo & Grahame, 2014) present a complex illustration of genomic architecture evolution during ecological speciation, revealing variable patterns of allelic differentiation and genome‐wide linkage during adaptation.

### 4.2 Divergence of trait‐associated loci

Though initially thought to house genes integral to speciation with gene flow, recent debate has arisen over alternative evolutionary and demographic mechanisms that also explain high genomic divergence (Cruickshank & Hahn, 2014; Ravinet et al., 2017). By using GWA tests for phenotypic traits with known roles in adaptive divergence between *L. saxatilis* ecotypes, we find evidence suggesting loci associated with divergence in shape between ecotypes show partial overlap with divergent genomic regions with elevated *F*_ST_ identified in Kess et al. (2018). These results indicate that selection on divergent shell morphology has likely contributed to genomic divergence between ecotypes in these regions, consistent with the hypothesis of genome‐wide divergence driven by ecological divergence (Via, 2012; Wu, 2001). These findings also reinforce the hypothesis that divergent selection rather than phenotypic plasticity underlies divergent shell morphology in *L. saxatilis* ecotypes inhabiting different intertidal regions (Hollander & Butlin, 2010). Our results differ from the findings of another recent genome‐wide association study of shell morphology conducted in Swedish *L. saxatilis* ecotypes, in which no individual significant loci at the genome‐wide level were identified (Westram et al., 2018). However, partitioning of morphological variance across chromosomes in the study by Westram et al. (2018) did uncover substantial phenotypic variation overrepresented in regions associated with structural variation (inversions) exhibiting elevated differentiation within the genome, consistent with our findings of differentiated QTL in the current study. These differences may arise due to methods of identifying associations between phenotypic and genomic variation, discussed below.

### 4.3 Genome‐wide association in populations with multiple sources of structure

We found more highly divergent SNPs underlying shell morphology divergence using polygenic RDA compared to single‐locus GWA models, which we attribute to differences in population structure corrections used in each method. Detection of trait‐associated loci using linear models that include principal components or a kinship matrix as covariates of population structure may be conservative when traits analyzed are strongly correlated with population structure (Atwell et al., 2010; Frichot, Schoville, Bouchard, & Francois, 2013). This problem may be more pronounced in scenarios of isolation‐by‐adaptation, in which local adaptation also reduces gene flow from other populations (Nosil, Egan, & Funk, 2008; Lotterhos & Whitlock, 2015), and has been observed in simulations studies (Forester et al., 2018).

Recent applications of multivariate ordination methods such as redundancy analysis have shown promise in finding multilocus adaptation while accounting for both population structure and polygenic interactions among loci (Capblancq, Luu, Blum, & Bazin, 2018; Forester et al., 2018). These methods have recently been shown to outperform mixed‐model‐based methods, as well as Random Forest, a machine‐learning‐based method, in uncovering loci associated with environmental variation (Capblancq et al., 2018; Forester et al., 2018). We acknowledge that although the RDA has performed better in simulation studies and appeared here to better match expectations from theory, the identification of a larger number of highly divergent trait‐associated loci does not guarantee that all these associations are true. Additionally, it is also possible that conservative correction for highly divergent loci in our single‐locus models also enabled the detection of polygenic loci that exhibit small shifts in allele frequency. Recent clinal analysis of loci exhibiting signatures of selection across Swedish ecotypes of *L. saxatilis* also exhibited variation in levels of differentiation, suggesting that there is a continuum of divergence observed among polygenic loci in this system (Westram et al., 2018). With these caveats in mind, all significant loci should be treated as hypotheses of trait association to be further investigated through gene annotation and crosses (Atwell et al., 2010; Savolainen, Lascoux, & Merilä, 2013).

### 4.4 Genomic architecture of trait‐associated loci

We find evidence suggesting that *L. saxatilis* shell shape exhibits linked, polygenic architecture predicted during trait divergence driven by genome‐wide differentiation (Feder, Egan, & Nosil, 2012), evidenced by identification of many candidate QTL with elevated LD. Observation of polygenic architecture is consistent with the previously identified quantitative genetic inheritance of shell shape in another *Littorina* species using half‐siblings reared at two different growth rates (Boulding & Hay, 1993). In comparing predicted genomic architectures expected under divergence with gene flow, observation of polygenic control of shape traits appears consistent with a scenario of multifarious selection resulting in the formation of statistically linked complexes of adaptive loci (Le Corre & Kremer, 2012; Flaxman et al., 2014). Consistent with this hypothesis, we observed elevated statistical linkage of QTL associated with shell morphology and exceeding levels observed in the neutral genome, indicating coupling of QTL both within and between ecotypes within each population. This pattern of coupling between loci associated with divergence is also predicted during adaptation and speciation with gene flow, resulting in elevated genome‐wide linkage during the buildup of reproductive barriers (Schilling et al., 2018). Buildup of genome‐wide LD during adaptive divergence has previously been identified in another model system for ecological speciation, threespine stickleback (Hohenlohe, Bassham, Currey, & Cresko, 2012). In models specific to *Littorinid* evolution, polygenic clines in shell thickness along a steep predation gradient were facilitated by positive LD among physically unlinked loci of small effect (Boulding et al., 2007). We observe elevated LD specifically at QTL, whereas the remainder of SNPs did not exhibit high LD between ecotypes (Table 3), suggesting that infrequent gene flow may still occur, despite a high level of overall neutral differentiation (Kess et al., 2018; Rolán‐Alvarez et al., 2004). We also uncovered more subtle patterns of within‐ecotype coupling of QTL, as predicted during evolution of locally adapted phenotypes (Schilling et al., 2018). We observed this pattern within at least one ecotype for each sampling location. Similar coupling of divergent loci within demes has also been identified across sympatric *Heliconius* host races and has been suggested to be important in enabling speciation (Schilling et al., 2018).

We did not find evidence of elevated physical linkage among QTL, as nearly all of these loci aligned to independent contigs across 17 linkage groups. Our finding is inconsistent with the recent identification of polymorphic inversions associated with formation of ecotypes across ecological gradients in *L. saxatilis* ecotypes (Faria, Johannesson, Butlin, & Westram, 2019; Morales et al., 2018), suggesting that these inversions may not be integral to shell shape divergence between ecotypes. Consistent with this observation, comparisons of whole‐genome divergence between pools of ecotypes have revealed also polygenic basis of ecotype divergence, although inversion regions did demonstrate elevated frequency of highly differentiated polymorphisms (Morales et al., 2018). The sparseness of RAD SNP loci (*N* = 4,066) in our dataset may have given us low coverage in inversion regions. Coupled with the level of fragmentation of the currently available reference genome, this limitation may explain why we did not find physical linkage among our QTL. The type II inversion hypothesis that balancing selection could maintain heterokaryotypes (Faria et al., 2019) should be further explored with whole‐genome resequencing data from individual snails matched with measurements of each individual's shells.

Also consistent with polygenic divergence of ecotype differentiation, we found predominantly loci of small effect contributing to shell morphology variation, but also identified a subset of large‐effect alleles in shell traits corresponding to ecotype survival and assortative mating. These larger effect size alleles (>10% phenotypic variation explained) were found in association with landmark locations related to shell aperture differences, consistent with the identification of variation in these traits between crab and wave ecotypes in the present study, as well as in past comparisons (Butlin et al., 2014; Carvajal‐Rodríguez et al., 2005). Large‐effect loci explaining more than 10% of the phenotypic variance in size were also observed. Size has traditionally been considered to possess a polygenic genetic architecture in other systems, though variation in effect size between associated loci has also been observed (Berenos et al., 2015; Visscher et al., 2007). Shell size has also been considered a “magic” trait in *L. saxatilis,* facilitating both reproductive isolation and adaptation to local predation and small biogenic refuges, respectively (Boulding et al., 2017; Servedio et al., 2011). Larger effect size of alleles underlying these traits may suggest stronger selection on these shell shape features, resulting in stronger selection for gene‐flow‐resistant trait architectures as well (Griswold, 2006; Albert et al., 2008). Given the role of shell size in controlling both local ecotype survival and assortative mating, identification of alleles of larger effect is consistent with models that predict large‐effect alleles incorporated into genetic architectures for traits underlying local adaptation with gene flow (Griswold, 2006; Yeaman & Whitlock, 2011).

Despite the discovery of loci of both large and small effect, sample size and genomic sampling method may both play a role in biasing estimates of allele number and effect size uncovered in this study. Our small sample sizes in both individuals and markers relative to massive human GWA studies may have prevented detecting the small contributions made to shell variation by many alleles dispersed across the genome (Boyle, Yang, & Pritchard, 2017) and may also bias effect size estimates of alleles detected (Otto & Jones, 2000). Additionally, RADseq samples a small subset of total genomic variation, potentially missing regions important to local adaptation (Lowry et al., 2017), although empirical results have largely supported the utility of RADseq in finding loci important to local adaptation (Catchen et al., 2017; McKinney, Larson, Seeb, & Seeb, 2017). Here, predicted genome size of *L. saxatilis* is 1.32 Gb (Vitturi, Libertini, Panozzo, & Mezzapelle, 1995), and we identify many QTL (216, from a small proportion of the surveyed genome (~0.01%). The nonrandom distribution of RAD loci within the genome (DaCosta & Sorenson, 2014), our selection of restriction enzymes that avoid repetitive regions, and our selection of a polygenic trait associated with genome‐wide divergence may account for this observation. Our identification of ~5.3% loci exhibiting trait association is similar to the proportion of divergent loci identified across other ecological speciation systems (Nosil et al., 2009) and, although not all QTL were identified as divergent outliers, is likely influenced by our selection of a trait crucial in ecotype divergence. Additionally, elevated LD between ecotypes and specifically at trait‐associated loci likely enabled detection of many QTL despite modest genomic coverage. Future studies that both employ larger samples and utilize whole‐genome sequences to sample the entirety of genomic variation provide a way to resolve this limitation.

## 5 CONCLUSIONS AND IMPLICATIONS

Our work here, using a genome‐wide SNP dataset and individual shell traits, paired with sampling across two glacial lineages, allowed us to uncover the genomic architecture of shell divergence in *L. saxatilis* crab and wave ecotypes across their range in northwest Spain. We used multiple models to discover the SNP markers associated with shell traits reflecting adaptations to differences in crab predation and wave action between regions of the intertidal zone. We detected polygenic trait architectures that overlapped regions of high genomic divergence as identified previously using *F*_ST_ outlier tests. We also identify elevated statistical linkage of identified genomic regions associated with shell traits despite no evidence of physical linkage, indicating that coordination of polygenic loci underlies ecotype formation in *L. saxatilis*. Future research on this system would benefit from the availability of a less‐fragmented genome that would improve the identification physical locations of QTL. It would also be interesting to associate gene expression data (e.g., Rivas et al., 2018) with the genome to facilitate identification of the causal trait‐associated SNP loci identified in this study.

## CONFLICT OF INTEREST

None declared.

## AUTHOR CONTRIBUTIONS

T.K. designed the project, conducted laboratory work and statistical analysis, and wrote the manuscript. E.G.B. helped design the project, supervised the laboratory work, assisted with interpretation of the statistical analysis, and helped write and edit the manuscript.

## Data Availability

Plink input files and shell phenotype data used for GWAS have been uploaded to Dryad (https://doi.org/10.5061/dryad.p3123d8). Reads will be uploaded to the NCBI Short Read Archive (SUB4134175).
